# The Neuroanatomical, Neurophysiological and Psychological Basis of Memory: Current Models and Their Origins

**DOI:** 10.3389/fphar.2017.00438

**Published:** 2017-06-30

**Authors:** Eduardo Camina, Francisco Güell

**Affiliations:** ^1^Mind-Brain Group: Biology and Subjectivity in Philosophy and Contemporary Neuroscience, Institute for Culture and Society, University of NavarraPamplona, Spain; ^2^Department of Learning and Curriculum, Faculty of Education and Psychology, University of NavarraPamplona, Spain

**Keywords:** explicit memory, sensory memory, implicit memory, long-term memory, short-term memory

## Abstract

This review aims to classify and clarify, from a neuroanatomical, neurophysiological, and psychological perspective, different memory models that are currently widespread in the literature as well as to describe their origins. We believe it is important to consider previous developments without which one cannot adequately understand the kinds of models that are now current in the scientific literature. This article intends to provide a comprehensive and rigorous overview for understanding and ordering the latest scientific advances related to this subject. The main forms of memory presented include sensory memory, short-term memory, and long-term memory. Information from the world around us is first stored by sensory memory, thus enabling the storage and future use of such information. Short-term memory (or memory) refers to information processed in a short period of time. Long-term memory allows us to store information for long periods of time, including information that can be retrieved consciously (explicit memory) or unconsciously (implicit memory).

## Introduction

A life full of unconnected events, of errors that do not lead to any lessons and of emotions without the ability to remember them is no life at all. Memory is precisely the capacity that allows us to connect experiences, learn and make sense of our lives. In short, it allows us to build our story. The full range of this complex capacity’s neuroanatomical, neurobiological, neurophysiological, and psychological mechanism remain unknown and it presents a challenge for psychologists and neuroscientists who try to explain it. This review attempts to provide a rigorous overview that permits anyone who wants to approach the latest scientific findings on memory to do so, as well as to understand them and properly order them. We will focus on neuroanatomical, neurophysiological, and psychological mechanisms of the different types of memory.

Although knowledge of molecular mechanisms is important for constructing a complete vision of memory models, in this article we can only point out general traits as summarized in this introduction [for more information see (Kandel et al., 2014)]. In addition, knowledge gained from neuroimaging studies (Binder and Desai, 2011), as well as knowledge of the neural markers associated with memory (Meneses, 2015), will likely play a key role in future models of memory mechanisms, but in this review, as stated above, we focus mainly on neuroanatomical, neurophysiological, and psychological mechanisms.

We believe it is important to consider previous developments without which one cannot adequately understand the classifications of memories and the kinds of memory models that are now current in the scientific literature.

The three major classifications of memory that the scientific community deals with today are as follows: sensory memory, short-term memory, and long-term memory. Information from the world around us begins to be stored by sensory memory, making it possible for this information to be accessible in the future. Short-term memory refers to the information processed by the individual in a short period of time. Working memory performs this processing. Long-term memory allows us to store information for long periods of time. This information may be retrieved consciously (explicit memory) or unconsciously (implicit memory).

As Squire (2004) points out, the first theoretical approaches relevant to current neuroscience come from the 19th century. These include Maine de Biran (1804/1929) (Maine de Biran, 1929) who, at the beginning of the century, wrote of mechanical memory, sensitive memory, and representative memory. The philosopher James, and his book *The Principles of Psychology* (James, 1890), is also especially worth highlighting. Therein, James distinguishes between primary and secondary memory, thereby referring to short- and long-term memory, respectively.

The importance of Pavlov (1927) and Fitts and Posner (1967) are especially noteworthy during the first two thirds of the 20th century. Pavlov’s studies are related to a type of memory that later would be called associative memory. Meanwhile, Fitts and Posner’s studies are considered the first model to explain procedural memory.

Prior to the 60’s, most systematizations of memory distinguished a more mechanical type of memory related to the acquisition of skills, which is, in turn, related to activity of the intellect. Unlike what followed, debates in this period were mainly philosophical or based on psychological intuition (Ribot, 1881; Korsafoff, 1890).

Beginning in the 1960s, a series of experimental studies on how the brain stores information emerged, using animals and amnesic patients. Within this decade, Milner, Atkinson, and Shiffrin were especially important researchers.

The experimental modern era arguably began when Milner (1962) demonstrated, with HM experiments, that a seriously ill patient could acquire a new skill (hand-eye coordination) without any memory of having encountered the task before. “While this finding showed that memory is not unitary, discussions at the time tended to set aside motor skills as a special case representing a less cognitive form of memory. The suggestion was that the rest of memory is of one piece and is dependent on medial temporal lobe structures” (Squire and Wixted, 2016).

A few years later, Atkinson and Shiffrin (1968) proposed a modal model of memory that constitutes one of the most influential explanations for the existence of different components in the memory system. The importance of this model is such that it must be explained in the next section, but for now it should simply be mentioned that the modal model establishes the existence of short-term storage (ACP), which receives sensory information that is processed by sensory and data storehouses within long-term memory. This storage system can generate reasoning and new deductions from existing ones.

In the seventies, Tulving, Baddeley, and Hitch and Kandel’s investigations are especially noteworthy. Tulving (1972) first proposed the distinction between episodic memory and semantic memory. Baddeley and Hitch (1974) conducted research on the components of working memory. Both authors considered working memory as a limited capacity system that allows temporary storage and manipulation of information necessary to perform complex tasks such as understanding, learning, and reasoning. As explained later on, at first (1974), they proposed the existence of three subsystems within the multi-storehouse model of short-term memory: the central executive, a phonological or articulatory loop and a visuospatial sketchpad. Later, Baddeley (2000) included a fourth subsystem, the episodic buffer, which combines information from the subsystems in a form of temporal representation. Kandel (1976) proposed a model to explain the mechanism of operation in habituation and sensitization. To do this, he used the notion of non-associative memory, which, as we shall see, is one of the four types of non-declarative or implicit memory, like that which refers to new behaviors learned through repeated exposure to a single stimulus. According to Kandel, new behaviors can be classified into two processes: sensitization and habituation. On the one hand, for habituation, acetylcholine is progressively consumed, decreasing the effectiveness of the stimulus and thereby the motor response. On the other hand, the presence of serotonin in sensitization, secreted by another sensory nerve terminal, causes an excess of acetylcholine. An enhanced motor response thus emerges.

In the 1980s, the differences between declarative and non-declarative memories were consolidated and disseminated. This, together with contributions from Tulving and others, such as Di Lollo or Graf and Schacter, enabled a more precise classification of different types of memory. To date, Di Lollo’s model of iconic memory (Di Lollo, 1980) has been the most widely accepted and studied of the three existing types of sensory memory. As discussed in the next section, Di Lollo considered iconic memory a storage unit consisting of two components: the persistence of vision and information. Graf and Schacter (1985) proposed a general difference between declarative memory (explicit) and non-declarative memory (implicit/procedural). This stems from the distinction that Tulving (1972) proposed between the aforementioned episodic memory and semantic memory (both, as we will see, are currently included in declarative memory).

In the 90’s, a classification of the types of memory emerged, but the way they act and their interrelation was still unclear. In order to clarify its operation, Packard and McGaugh (1996) proposed that memory systems operate independently and in parallel. For example, an adverse event in childhood (e.g., seeing your grandfather being run over by a combine) can, on the one hand, consolidate as a stable declarative memory for the event itself (the sound of a combine always makes you remember that moment-episodic memory) and, on the other hand, can crystallize in non-declarative memory and result in a phobia experienced as a personality trait rather than as a mere memory (being near a combine will always produce panic and induces a desire to escape that situation-associative memory). Several authors (Tulving et al., 1982) had already mentioned the idea of priming as a separate type of memory, but it was not until the 90’s that experiments were conducted to show it (Hamann and Squire, 1997; Stark and Squire, 2000; Levy et al., 2004). These studies show that severely amnesic patients can exhibit completely intact priming while performing memory tests that include conventional recognition of the same test items (Squire, 2004).

Thanks to the development of new 21st century technologies, researchers have been able to more accurately locate brain areas that are associated with different types of memory. Although this pertains to topics to be addressed in detail in the next section, there are two examples that we consider significant to the application of these new techniques and the significant progress made in understanding memory storage. On the one hand, Ergorul and Eichenbaum’s experiment (Ergorul and Eichenbaum, 2004) shows that animals are able to remember the “context in which they experienced specific stimuli, and that this capacity also depends on the hippocampus” (Dickerson and Eichenbaum, 2010). This process is closely related to the formation of episodic memory. On the other hand, neuroimaging studies that Binder and Desai (2011) conducted show “two striking results: the participation of modality-specific sensory, motor, and emotion systems in language comprehension, and the existence of large brain regions that participate in comprehension tasks but are not modality-specific.” With this in mind, Binder and Desai (2011) claims that semantic memory consists of two representations, including a specific mode and a supramodal mode. Again, this will be explained in more detail in what follows.

The research of the cellular and molecular substrates of memory has received much attention since Lomo (1966) described in the 60’s “a cellular model of experience-dependent plasticity—long-term potentiation (LTP)” (Kandel et al., 2014). According to Lisman et al. (2012): “LTP is a process whereby brief periods of synaptic activity can produce a long-lasting increase in the strength of a synapse, as shown by an increase in the size of the excitatory postsynaptic current.” NMDA receptors are double-gated, as their activation requires both postsynaptic membrane depolarization as well as presynaptic release of glutamate. Once activated by these conditions, NMDA receptors trigger a strong postsynaptic influx of Ca2+ that induce LTP through a variety of pathways including CaMKII, PKC, PKA, and MAPK (Kandel et al., 2014).

With this brief historical and conceptual introduction laid out, we intend to delve into different types of memory in order to present the models that the scientific community has most accepted thus far. In the last section, and before the glossary, we identify the likely directions for future research. Now we turn on to our main task, presenting an overview of the latest scientific findings on memory, classified according to different types and mechanisms.

## Sensory Memory: Iconic Memory

“Sensory memory is the capacity for briefly retaining the large amounts of information that people encounter daily” (Siegler and Alibali, 2005). There are three types of sensory memory: echoic memory, iconic memory, and haptic memory. Iconic memory retains information that is gathered through sight, echoic memory retains information gathered through auditory stimuli and haptic memory retains data acquired through touch.

Scientific research has focused mainly on iconic memory; information on echoic and haptic memory is comparatively scarce. Thus, taking into account the goals of this article and that it is aimed at a higher education audience, presenting iconic memory as a paradigm of sensory memory is sufficient for an introductory overview.

Iconic memory retains information from the sense of sight with an approximate duration of 1 s. This reservoir of information then passes to short-term vision memory (which is analogous, as we shall see shortly, to the visuospatial sketchpad with which working memory operates).

Di Lollo’s model (Di Lollo, 1980) is the most widely accepted model of iconic memory. Therein, he considered iconic memory a storehouse constituted by two components: the persistence of vision and information.

(a) Persistence of vision. Iconic memory corresponds to the pre-categorical representation image/visual that remains between 100 and 300 ms. It is sensitive to physical parameters, such that it depends on retinal photoreceptors (rods and cones). It also depends on various cells in the visual system and on retinal ganglion cells M (transition cells) and P (sustained cells). It concludes its representation in the primary visual cortex (V1) of the occipital lobes. “The occipital lobe is responsible for processing visual information” (Kamel and Malik, 2014).

(b) Persistence of information. Iconic memory is a storehouse of information that lasts 800 ms and that represents a codified and already categorized version of the visual image. It plays the role of storehouse for post-categorical memory, which provides visual short-term memory with information to be consolidated. For this, it travels through the ventral route (V) (V1 → V2 → V5 → inferior temporal cortex).

Subsequent research on visual persistence from Coltheart (Coltheart, 1983) and Sperling’s studies (Sperling, 1960) on the persistence of information led to the definition of three characteristics pertaining to iconic memory: a large capacity, a short duration, and a pre-categorical nature.

Sperling (1960) demonstrated this large capacity after presenting the results of his total and partial reports. The full report consisted in presenting a 3 × 3 or 3 × 4 matrix of alphanumeric characters for a short period of time to subjects and later asking them which characters they remembered. On the other hand, in the partial report, subjects were directed to remember the characters in a row specifically assigned to them in the instructions. The total report’s results showed that subjects were only able to recall between 3 or 4 letters of the total number. However, in the partial report, subjects remembered around 75% of those that were asked. In extrapolating the partial report’s data to the total, it follows that individuals could report 9 of the 12 letters contained in the instructions (80% of the total), thus demonstrating a large capacity.

Regarding short-term, Sperling interpreted the results of the partial report as due to the rapid decline of the visual sign and reaffirmed this short duration by obtaining a decrease in the number of letters reported by the subject in delaying the audio signal for choosing a row to remember in the presentation. Averbach and Coriell’s experiments (Averbach and Coriell, 1961) corroborated Sperling’s conclusion; they presented a variety of letters for a certain period of time to the subject. After each letter, and in the same position, they showed a particular visual sign. The participant’s task was to name the letter that occupied the position of the visual sign. When the visual sign appeared immediately after the letters, participants could correctly name the letter that occupied the position of the sign, however, as the presentation of the sign became more delayed, participant performance worsened. These results also show the rapid decline of visual information.

Finally, regarding its pre-categorical nature, Sperling considered the information contained in this storehouse as physical information that maintains the raw data that is not related to the meaning of stimuli. Subsequently, evidence has been obtained that this system is not entirely pre-categorical (Loftus et al., 1992) since the task improves when the stimuli to remember are letters or numbers instead of meaningless forms.

## Short-Term Memory

**FIGURE 1:**
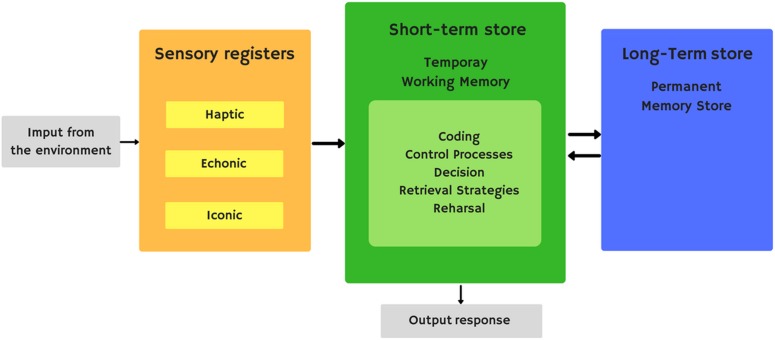
Atkinson and Shiffrin’s modal model.

Short-term memory is the ability to keep a small amount of information available for a short period of time. Atkinson and Shiffrin’s modal model (Atkinson and Shiffrin, 1968) is one of the most influential explanations for the existence of different components in the memory system (**Figure 1**). This model has some similarities with Broadbent’s previous model (Broadbent, 1958). The modal model establishes the existence of a short-term storehouse with limited capacity. The short-term storehouse receives sensory information processed by sensory storehouses and data in long-term memory. In addition, the short-term storehouse can also send information to the structures involved in long-term memory. This storehouse can generate reasoning and new deductions from existing ones. This model implies that the short-term storehouse functions as a kind of working memory, a system to retain and manipulate information temporarily as part of a wide range of essential cognitive tasks such as learning, reasoning, and understanding. They, in turn, give short-term storage central importance in the overall processing of information by attributing to it the role of controlling the executive system, responsible for the coordination and control of many complex subroutines in charge of acquiring new material and recovering old material in long-term storage.

Despite the explanatory power of Atkinson and Shiffrin’s model, there were a number of issues that this model could not resolve, causing criticism of it. For example, this model implies that the longer an item remains in memory, the more likely it is to be transferred to long-term storage. But studies like those of Tulving and Pearlstone (1966) and Craik and Watkins (1973) show that said relationship does not exist.

Given these criticisms, new models began to appear to explain memory, such as those from Cowan (1988, 1995, 1999) and Goldman-Rakic (1995). Among them, Craik and Lockhart’s process model (Craik and Lockhart, 1972) and Baddeley and Hitch’s structural model (Baddeley and Hitch, 1974) were the most prominent; the latter is the most commonly accepted one today and thus we will focus on it in this article.

As an introduction, it can be argued that Craik and Lockhart (1972) understood memory not as a process through which information is deepened at higher levels until it becomes part of long-term memory, but rather as a system of storehouses. Despite an emphasis on information processing (instead of structure), they continued to accept the existence of short-term memory as independent from long-term memory. For their part, Baddeley and Hitch’s proposal (Baddeley and Hitch, 1974) contemplated a multi-component working memory instead of a storage unit in the short term.

### Working Memory

“The term working memory refers to a brain system that provides temporary storage and manipulation of the information necessary for such complex cognitive tasks as language comprehension, learning and reasoning” (Baddeley, 1992). At first (1974), they proposed the existence of three subsystems within the multi-storehouse model of short-term memory: the central executive, a phonological or articulatory loop and a visuospatial sketchpad.

In general, we can say that the central executive controls attention, “the phonological loop ensures retention of verbal information and the visuospatial sketchpad is responsible for storage visual and spatial information” (Grigorenko et al., 2012). The latter two sub-memory systems are equivalent to verbal and visual short-term memory systems, respectively.

Wang and Bellugi (1994) presented a genetically based test that supports the functional and anatomical separation of Baddeley’s model with phonological and visuospatial storehouses. They compared two genetic syndromes (Williams and Down) with different brain morphology. Williams syndrome patients, despite having widespread mental handicaps, preserve their language skills, while Down syndrome patients preserve more partial capacities, but have very limited language skills. It was therefore assumed that the former would be better at verbal tasks related to operative memory, and that the latter would be better at visuospatial tasks related to operative memory. As expected, subjects with Williams syndrome performed better at phonological tasks, while subjects with Down syndrome, in turn, performed better at spatial tasks.

**FIGURE 2:**
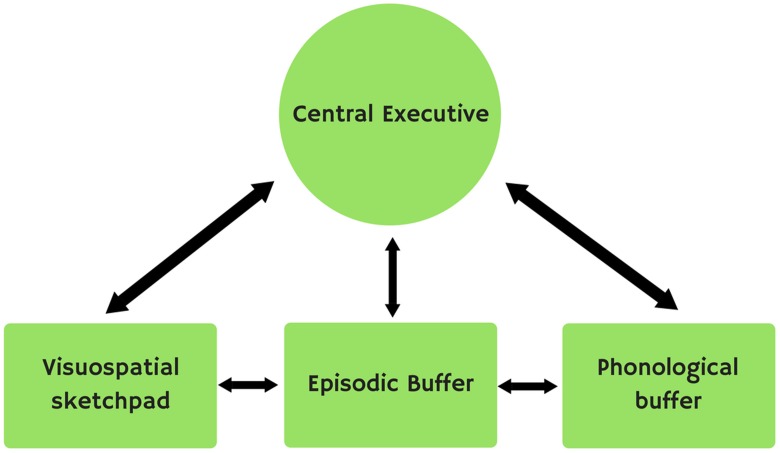
Baddeley’s model.

Later, Baddeley (2000) included a fourth subsystem, the episodic buffer (**Figure 2**), which combines information from the different subsystems in a kind of temporal representation.

Here we will focus on the different subsystems that make up Baddeley’s multi-storehouse model (2000), i.e., the central executive, the phonological or articulatory loop, the visuospatial sketchpad and the episodic buffer.

#### The Central Executive

The central executive is a system of attention control with limited processing capacity. Baddeley (1986) adopted a model originally proposed by Norman and Shallice (1986), in which actions are controlled in two ways. “Behavior that is routine and habitual is controlled automatically by a range of schemas, well-learned processes that allow us to respond appropriately to the environment” (Baddeley and Hitch, 2010). Processes that are not recognized as habitual are controlled by a second system, the supervisory attention system. This system uses long-term knowledge to propose novel behavioral solutions and to weigh options before deciding on a response.

In its original version, the central executive was considered an overall system capable of processing and storing. However, Baddeley and Logie (1999) proposed that it only has attention capacity.

Subsequent studies have proposed to complement the executive system with the episodic buffer as other separate storage system: “the episodic buffer clearly does represent a change within the working memory framework, whether conceived as a new component, or as a fractionation of the older version of the central executive”(Baddeley, 2000).

Baddeley and Logie understand the central executive as the result of the integration of several processes: the ability to focus attention, the ability to divide attention between two or more tasks, and the ability to control long-term memory access (Baddeley et al., 1991; Logie et al., 2004; Baddeley, 2007). The way to accomplish this may be with one or more types of inhibition (Engle et al., 1999; Miyake et al., 2000). This approach accepts that the frontal lobes play an important role in executive control, although there are differing opinions on the functions’ precise location (Duncan and Owen, 2000; Shallice, 2002).

#### Visuospatial Sketchpad

It has been suggested that the sketchpad’s main function is to create and maintain a visuospatial representation that persists through the irregular form found in eye movement and that characterizes our exploration of the visual world (Luck, 2007).

It has been shown that spatial tasks such as driving a car can interfere with spatial skills, while exclusively visual tasks, such as watching a series of images or colored shapes, can interfere with the recall of objects or shapes (Logie, 1986; Klauer and Zhao, 2004). These patterns of interference, together with cases of brain-damaged patients that show a deficit in one kind of task but not the other (Della Sala and Logie, 2002), suggest that spatial information and visual characteristics can be stored separately.

The visuospatial sketchpad seems to involve a number of areas, predominantly in the brain’s right hemisphere. On the one hand, it contains a visual component that reflects the processing and storage of objects and their visual features. On the other hand, it contains a second parietal area, presumably involved in spatial aspects.

#### Phonological Buffer

It can be argued that the phonological buffer supports language acquisition by providing the ability to store new words, while they are consolidated into long-term memory (Baddeley et al., 1998). Within this phonological loop, two basic sub-processes emerge: a short-term acoustic storehouse and a subvocal articulatory rehearsal process. The existence of the former is indicated by the effect of phonological similarity, where speech is less accurate when repeating “similar-sounding words such as MAN CAP CAT MAT CAN, than dissimilar words such as PIT DAY COW PEN TOP. Similarity of meaning (HUGE LARGE BIG WIDE TALL) has little effect on immediate recall. On the other hand if several trials are given to learn a longer list of say 10 words, meaning becomes all-important and sound loses it power, consistent with different systems for short-term and long-term storage (Baddeley, 1966a,b). Evidence for the importance of rehearsal comes from the word length effect, whereby immediate recall of long words (e.g., REFRIGERATOR UNIVERSITY TUBERCULOSIS OPPORTUNITY HIPPOPOTAMUS) is much more error-prone than for short words (Baddeley et al., 1975)” (Baddeley and Hitch, 2010). Baddeley and Hitch (1974) proposed that retention of items in the short-term storehouse quickly fade, but can be maintained by repeating them.

With respect to cerebral location, the phonological loop is found in the brain’s left hemisphere “The loop is assumed to hold verbal and acoustic information using a temporary store and an articulatory rehearsal system, which clinical lesion studies, and subsequently neuroradiological studies, suggested are principally associated with Brodmann areas, 40 and 44, respectively” (Baddeley, 2000).

#### Episodic Buffer

The verbal and visual systems within the conventional model of working memory may explain many aspects, but Baddeley (2000) points out that evidence from patients with short-term memory deficits— who resist memorizing prose (with a verbal span much higher than that of isolated words) and resist serial memory of articulatory suppression— leads to supposing that a storehouse of additional support exists. This is seen in the existence of a new mechanism that combines information from multiple subsystems into a form of temporal representation. Baddeley (2000) proposed the term episodic buffer for this new kind of representation.

The episodic buffer is thus a temporary storage system capable of integrating information from different sources, likely controlled by the central executive. “The buffer is episodic in the sense that it holds episodes whereby information is integrated across space and potentially extended across time” (Baddeley, 2000). It can be preserved in patients with advanced amnesia and severe impairment of long-term episodic memory.

With that said, it is possible to consider the episodic buffer as conceptual short-term memory. Studies to date do not specify activity in a specific area. As Potter (1999) said: “The conceptual short-term memory hypothesis proposes that when a stimulus is identified, its meaning is rapidly activated and maintained briefly in conceptual short-term memory.”

## Long-Term Memory

Long-term memory refers to unlimited storage information to be maintained for long periods, even for life. There are two types of long-term memory: declarative or explicit memory and non-declarative or implicit memory.

Explicit memory refers to information that can be consciously evoked. There are two types of declarative memory: episodic memory and semantic memory. For its part, implicit memory encompasses all unconscious memories, such as certain abilities or skills. There are four types of implicit memory, including procedural, associative, non-associative, and priming.

### Declarative/Explicit Memory

Explicit memory refers to information that can be evoked consciously. There are two types of declarative memory: episodic memory and semantic memory. As shown below, episodic memory stores personal experiences and semantic memory stores information about facts.

#### Episodic Memory

“Episodic memory involves the ability to learn, store, and retrieve information about unique personal experiences that occur in daily life. These memories typically include information about the time and place of an event, as well as detailed information about the event itself.” (Dickerson and Eichenbaum, 2010).

There are a number of neural components that are closely related to the proper functioning of episodic memory, which include the following: the cortex near the hippocampus [as discussed below, the perirhinal cortex (PRC), the entorhinal cortex, and the parahippocampal cortex (PHC)], cortical and subcortical structures, and the circuits within the medial temporal lobe and hippocampus.

The cortices near the hippocampus extensively interact with a number of cortical and subcortical structures; cortical components have key roles in various aspects of perception and cognition, while the medial temporal lobe structures mediate the organization and the persistence of the memory network, whose data is stored in these cortical areas (Dickerson and Eichenbaum, 2010).

The structures directly related to the hippocampus include the entorhinal, the parahippocampal, and the perirhinal cortices. Each one is discussed in detail below.

The entorhinal cortex is the main interface between the hippocampus and neocortex, thus it is associated with the distribution of information to and from the hippocampus. The surface layers (II and III) of the entorhinal cortex project out toward the dentate gyrus and hippocampus. While layer II mainly projects out toward the dentate gyrus and the CA3 region of the hippocampus, layer III mainly projects out toward the hippocampal CA1 region and the subiculum. These layers receive input signals from other cortical areas, particularly the association cortices, the PRC and the parahippocampal gyrus, as well as the prefrontal cortex. Layers II and III receive highly processed inputs from each sensory modality, and inputs related to ongoing cognitive processes. Deep layers, particularly layer V, receive one of the three output signals from the hippocampus and, in turn, exchange connections with other cortical areas that project out toward the superficial entorhinal cortex.

The PRC has a role in visual object recognition, while the PHC is involved in the perception of the local environment and processing information related to that place. Thus, fMRI studies indicate that the PHC becomes very active when human subjects receive topographical stimuli such as landscapes or rooms. Epstein and Kanwisher (1998) first described the PHC and Aguirre et al. (1996, 1998) and Ishai et al. (1999) later backed up that description.

Finally the hippocampus is responsible for the formation and retrieval of memories. That is, the information that the three cortices described above process reach the hippocampus where new memories are generated and from which they can later be retrieved. Episodic memory recall involves a spatial and temporal context of specific experiences. For further review of the mechanisms of memory formation see Craver (2003).

As Dickerson and Eichenbaum (2010) point out in their review, “several investigators have argued that animals are indeed capable of remembering the context in which they experienced specific stimuli, and that this capacity also depends on the hippocampus.” Ergorul and Eichenbaum (2004) published a significant study to this effect in which they developed a series of tasks for rats to assess their memory of events, which combined an odor (what), the place of the experience (where), and the relation to other experiences (when). The rats were presented with a sample of an odor in one specific place along the edge of a large open field. Subsequently, as a way of testing their memory, they were presented with a choice between two arbitrarily selected odors in their original locations. The results of the test showed that normal rats use a combination of where and what information to judge the timing of the events, while rats with a damaged hippocampus cannot manage to effectively combine what, when, and where information in order to form a recovered memory.

Three years later Eichenbaum et al. (2007) proposed a functional organization of memory’s medial temporal lobe system: “Neocortical input regarding the object features (“what”) converges in the PRC and lateral entorhinal area (LEA), whereas details about the location (“where”) of objects converge in the PHC and medial entorhinal area (MEA). These streams converge in the hippocampus, which represents items in the context in which they were experienced. Reverse projections follow the same pathways back to the parahippocampal and neocortical regions”(Eichenbaum et al., 2007).

It should be noted that memory of faces is typically associated with activity in the perirhinal and hippocampus rostral regions, while memory of objects is typically associated with wider-ranging activity (Preston et al., 2010).

Both results concerning functional an anatomic and characterizations in animal models are consistent with the hypothesis that is guided by anatomic criteria about the functional organization of the hippocampal system (Dickerson and Eichenbaum, 2010).

“The ventral temporal cortex, including fusiform gyrus, is commonly engaged when pictures of visual objects are presented, and the lateral temporal cortex including superior temporal gyrus is typically engaged during the encoding of auditory information” (Dickerson and Eichenbaum, 2010).

#### Semantic Memory

As noted, in the context of long-term memory, there were two types of memory, corresponding to declarative and non-declarative memory. Within declarative memory, we find both episodic memory, as discussed above, and semantic memory, as discussed below.

Human beings have the ability to represent concepts in language. This ability allows us not only to disseminate conceptual knowledge to others, but also to manipulate, associate, and combine these concepts. Therefore, as Binder and Desai shows, “humans use conceptual knowledge for much more than merely interacting with objects. All of human culture, including science, literature, social institutions, religion, and art, is constructed from conceptual knowledge” (Binder and Desai, 2011). Activities such as reasoning, planning for the future or reminiscing about the past depend on the activation of concepts stored in semantic memory (Mahon and Caramazza, 2008).

Binder and Desai showed two striking results related to neuroimaging research: on the one hand, the participation of the specific sensory, motor and emotional modality in understanding language and, on the other hand, the existence of large regions of the brain (the inferior parietal lobe and a large part of the temporal lobe) involved in tasks related to understanding. These latter regions converge on the many currents involved in perception processing, and these convergences allow supramodal representations of perceptual experience that support a variety of conceptual functions, including language, social cognition, object recognition, and the extraordinary human ability to remember the past and imagine the future (Binder and Desai, 2011). Therefore, accepting their argument, semantic memory consists of two representations: a specific modality and supramodal modality.

In this regard, Binder and Desai found several objections. A not inconsiderable one is that activations observed in imaging experiments could be an epiphenomenon rather than causally related to understanding. Therefore, the involvement of the motor system for processing a text would contribute to understanding and is not a mere product. Another critical point is the possibility of interpreting that collected activations represent images after understanding takes place. However, and as they showed in their review (Binder and Desai, 2011), in studies of neuroimaging with high temporal resolution, the activation of motor regions during the processing of a text appears to be rapid, about 150–200 ms after each word (Pulvermüller et al., 2005; Boulenger et al., 2006; Hoenig et al., 2008; Revill et al., 2008).

“These converging results provide compelling evidence that sensory-motor cortices play an essential role in conceptual representation. Although it is often overlooked in reviews of embodied cognition, emotion is as much a modality of experience as sensory and motor processing (Vigliocco et al., 2009). Words and concepts vary in the magnitude and specific type of emotional response they evoke, and these emotional responses are a large part of the meaning of many concepts” (Binder and Desai, 2011).

Following Binder and Desai, brain appears to use supramodal abstract representations for conceptual tasks. In this regard, it can be convincingly argued that the human brain has large areas of cortex that are between the sensory systems and motor modalities and, therefore, Damasio’s idea convergence zones seems plausible (Damasio, 1989). “These heteromodal areas include the inferior parietal cortex (angular and supramarginal gyri), large parts of the middle and inferior temporal gyri, and anterior portions of the fusiform gyrus (Mesulam, 1985)” (Binder and Desai, 2011).

A second argument supporting the hypothesis that the brain appears to use supramodal abstract representations during conceptual work comes from patients with damage to the lower and lateral temporal lobe. The clinical profile of semantic dementia is marked by progressive atrophy in the temporal lobe and loss of multimodal semantic memory (Hodges et al., 1992; Mummery et al., 2000). Patients with semantic dementia is characterized by a loss of conceptual knowledge, and this loss may reflect the disruption of a central semantic hub or the degeneration of a temporosylvian language network for verbal concepts (Irish et al., 2014). These patients manifesting in striking alterations in naming and comprehension (Irish et al., 2016). These patients are “characterized by a clear dissociation between marked single-word comprehension” (Agosta et al., 2010), unable to retrieve the names of objects, irregular word reading deficits, identify the color the correct objects, and sparing of fluency, phonology, syntax and working memory (Binder and Desai, 2011).

Basically, these deficits do not seem to be categorical, constituting further evidence that semantic impairment does not imply strongly modal representations and, therefore, the modular and supramodal systems are presented as an interactive continuum of hierarchically ordered neuronal combinations, supporting representations that are progressively more idealized and combined (Binder and Desai, 2011). These systems correspond to Damasio’s idea of areas of local convergence and with Barsalou’s idea of systems of unimodal perceptual symbols (Lambon Ralph et al., 2007). In addition to bottom-up input within their associated modality, each system receives top-down input from other modal and attention systems. These systems are modal in the sense that their output is a analogic or isomorphic representation of the information that they receive bottom-up within their associated modality (Barsalou, 1999a).

As observed by Binder and Desai: “These modal convergence zones then converge with each other in higher-level cortices located in the inferior parietal lobe and much of the ventral and lateral temporal lobe (…). One function of these high-level convergences is to bind representations from two or more modalities, such as the sound and visual appearance of an animal, or the visual representation and action knowledge associated with a hand tool (Wernicke, 1974; Damasio, 1989; Barsalou, 1999b; Patterson et al., 2007). Such supramodal representations capture similarity structures that define categories, such as the collection of attributes that place ‘pear’ and ‘light bulb’ in different categories despite a superficial similarity of appearance, and ‘pear’ and ‘pine-apple’ in the same category despite very different appearances (Rogers and McClelland, 2004). More generally, supramodal representations allow the efficient manipulation of abstract, schematic conceptual knowledge that characterizes natural language, social cognition, and other forms of highly creative thinking (Dove, 2011; Diefenbach et al., 2013)” (Binder and Desai, 2011).

### Non-declarative/Implied Memory

As noted, long-term memory refers to unlimited information storage that can be maintained for long periods, even for life. There are two types of long-term memory: declarative or explicit memory and non-declarative or implied memory.

Implicit memory encompasses all unconscious memories, as well as certain abilities or skills. There are four types of implicit memory: procedural, associative, non-associative, and priming. Each one is detailed below.

#### Procedural Memory: Habits and Skill

Procedural memory is the part of memory that participates in recalling motor and executive skills that are necessary to perform a task. It is an executive system that guides activity and usually works at an unconscious level. When necessary, procedural memories are retrieved automatically for use in the implementation of complex procedures related to motor and intellectual skills.

Development of these rote capacities occurs through procedural learning, that is, by systematically repeating a complex activity until acquiring and automatizing the capacity of all neural systems involved in performing the task to work together.

The acquisition of skills requires practice. However, the simple repetition of a task does not ensure skill acquisition. A skill is thought to be acquired when behavior changes as a result of experience or practice. This is known as learning and it is not a directly observable phenomenon. Here we will discuss two models for acquiring skills.

The first model comes from Fitts’s team (Fitts, 1954; Fitts and Posner, 1967). These scientists propose an explanatory model of skill acquisition, based on the idea of learning as a process in three phases:

(a) Cognitive phase: The process begins with the acquisition of knowledge about the factors that make up a particular observed behavior. At this point, the psychological process of attention is important. The skill to be acquired must be broken down into its basic components and one must understand how these components are combined to form a whole in the correct execution of the task (Fitts, 1954; Fitts and Posner, 1967).
(b) Associative phase: Individual repeated practice takes place until there is an automatic response pattern. As one progresses through this point, the actions that are important for the implementation of a skill are learned and become automated, just as any superfluous or ineffective actions disappears. The individual sensory system acquires the exact symbolic and spatial data required for the appropriate execution of the skill (Fitts, 1954; Fitts and Posner, 1967).
(c) Autonomous/procedural phase: This is the final phase and it consists in perfecting acquired skills. The ability to judge which stimuli are important and unimportant improves and a lower level of conscious thought is required because the skill becomes automated (Fitts, 1954; Fitts and Posner, 1967).

The other model corresponds to (Tadlock, 2005) and is called Predictive Cycle. This model proposes that learning only requires conscious maintenance of the desired end result. The model consists of the following phases: Trial, error, implicit result analysis, and decision-making at the implicit level of the way in which execution of the next test must be changed for successful implementation. These steps are repeated again and again until the subject builds or remodels his/her neural network so that it can guide the activity without the need for conscious thought.

A number of factors are involved when acquiring and implementing skills, including attention and pressure. For the acquisition of a new skill one must pay attention to the steps to be undertaken. This process involves using working memory to allow for connecting the different steps involved. Procedural memory acquires the habit with the help of the attention span, but it implies a lesser performance. However, with practice, procedural knowledge is developed. Procedural knowledge operates away from working memory, which allows for the implementation of the most automated skills (Anderson, 1993). Meanwhile, pressure can affect the performance of a task in two ways: choking or clutchness. The choking phenomenon occurs when experienced and skilled performers fail under stress. Auto-focus theories suggest that pressure causes an increase in anxiety and self-consciousness concerning correct execution. This ends up causing increased attention directed toward processes directly involved in the execution of the skill (Beilock and Carr, 2001). On the other hand, the attention span allows the habit to be acquired in refers to giving a top performance on a given task when pressure is highest.

Because they are especially relevant, we will briefly outline brain components involved in the acquisition of new skills and habits, including the basal ganglia, cerebellum and limbic system.

As Christos and Emmanuel explain (Constantinidis and Procyk, 2004), “basal ganglia are formed by several sub-structures: the striatum, the globus pallidus, the substantia nigra, and the subthalamic nucleus.” The basal ganglia are a collection of nuclei found on both sides of the thalamus, outside of and around the limbic system, but below the cingulate gyrus and within the temporal lobes. The striatum or striate nucleus is the main gateway for information to the basal ganglia. In turn, the striatum receives information from the cerebral cortex. Essentially, there are two parallel processing paths that depart from the striatum, each of which acts in opposition to each other in the control of movement and enables associations with other relevant functional structures (Beilock and Carr, 2001). Both work together as a neuronal feedback loop. There are many circuits that reach the striatum from other brain areas, including the limbic cortex (associated with emotional processing); the ventral striatum (related to the processing of rewards), and other important motor regions involved in movement (Alexander and Crutcher, 1990). Currently, striatal neuronal plasticity enables basal ganglia circuits to interact with other structures and thereby contribute to the processing of procedural memory (Haber et al., 2000).

The cerebellum is involved in the execution of movements and the perfection of motor agility needed procedural skills. Damage to this area can impede one from relearning motor skills and recent studies have linked it to the process of automating unconscious skills during the learning phase (Kreitzer, 2009).

The limbic system shares anatomical structures with a component of the neostriatum, which assumes primary responsibility for the control of procedural memory. There is a special protein membrane associated with the limbic system that runs through the nucleus basalis. Thus, activation of brain regions that work together during the operation of procedural memory can be followed through the protein membrane associated with the limbic system.

As a final note on procedural memory, whereas earlier theories proposed a passive role whereby memories were shielded from interfering stimuli during sleep (Vertes and Eastman, 2000; Vertes, 2004), current theories suggest a more active role in which memories undergo a process of consolidation during sleep (Ellenbogen et al., 2006). Furthermore, in human beings, this process of consolidation is thought to contribute to the development of procedural knowledge, especially when it occurs right after the initial phase of memory acquisition (Karni et al., 1994; Gais et al., 2000; Stickgold et al., 2000a,b; Saywell and Taylor, 2008). Within the scope of motor skills related to procedural memory, there is evidence to show that there is no improvement in skills if followed by short NREM sleep (stages 2–4 sleep), such as a short nap (Walker et al., 2002). However, REM sleep (a sleep phase with an increased frequency and intensity of the so-called dream state) followed by a period of slow wave sleep has proven to be the most effective combination for procedural memory consolidation, especially immediately following skill acquisition (Siegel, 2001).

#### Associative Memory: Classical and Operant Conditioning

Associative memory refers to the storage and retrieval of information through association with other information. The acquisition of associative memory is carried out with two types of conditioning: classical conditioning and operant conditioning. Classical conditioning is associative learning between stimuli and behavior. Meanwhile, operant conditioning is a form of learning in which new behaviors develop in terms of their consequences. Associationist philosophers have also worked with the latter model (Hartley, 1749; Mill, 1829). We will look more closely at both.

The close association between two stimuli over time causes classical conditioning: first a conditioned stimulus and then an unconditioned stimulus. While a conditioned stimulus does not automatically trigger a response, an unconditioned stimulus does just that. By repeating a conditioned stimulus over time before an unconditioned stimulus, a conditioned stimulus acquires characteristics that simulate being necessary for an unconditioned stimulus. Pavlov’s Dog (Pavlov, 1927) is a clear example. The dog produces saliva when it detects the presence of food (unconditioned stimulus). If the sound of a bell goes off (conditioned stimulus) during the act of giving the dog food, the dog will associate the sound of the bell with the presence of food. In successively repeating this, the dog will associate the unconditioned stimulus with the conditioned stimulus, thus producing saliva when just hearing the bell.

Although Skinner is considered to be the originator of operant conditioning, his research drew upon Thorndike’s law of effect. For operant conditioning, as has already been mentioned, positive consequences following a behavior promote its repetition. Conversely, if the behavior involves negative consequences, the behavior will be repeated less. Thorndike (1932) called this conditioning instrumental because it suggests that the behavior serves as a means to an end and emerges from trial and error. Skinner later coined the term that is now widely associated with this law of effect – reinforcement (Skinner, 1938).

#### Non-associative Memory: Habituation and Sensitization

Non-associative memory is one of three types of non-declarative or implicit memory and refers to newly learned behavior through repeated exposure to an isolated stimulus.

New behavior can be classified into two processes: sensitization and habituation (Alonso, 2008). Before delving into each process, it is worth noting that the simplicity in acquiring this type of memory has advanced knowledge of the learning process. This is due to the fact that both animals and human beings have these two processes, such that it is very likely that, in this regard, they share a molecular biological basis. Kandel (1976) proposed a model to explain habituation and sensitization’s operation mechanism. On one hand, for habituation, acetylcholine is progressively consumed, decreasing the effectiveness of the stimulus and thus the motor response. Furthermore, for sensitization, the presence of serotonin, secreted by another sensory nerve terminal, causes an excess of acetylcholine. Thus an enhanced motor response emerges. Let’s look at the two processes that take place in the acquisition of new behaviors— processes that are part of non-associative memory, habituation and sensitization.

Habituation, in this context, is linked to repetition. The repetition of a stimulus leads to a decrease in its response, which is known as habituation. Repeated exposure to a stimulus serves to stop responding to potentially important, but situationally irrelevant stimuli. Habituation could be due to a process of synaptic depression as a result of repeated activation. Thus, habituation is thought to be related to a decrease in the efficiency of synaptic transmission, a decrease that may be caused by a conductivity change in the membrane of the stimulated neuron’s iconic channels.

Unlike habituation, sensitization consists in an increase in response to a stimulus due to the repeated introduction thereof. Although the processes that produce sensitization are the same as those that produce habituation, sensitization’s effects are the opposite since it results in an increase of the original response. The process of sensitization may be due to a provision in transmission, whether it be presynaptic or postsynaptic.

#### Priming

Priming, the fourth modality of non-declarative or implicit memory, is an effect whereby exposure to certain stimuli influences the response given to stimuli presented later.

An example is in order. If you present a list of words to a person that contains the word ‘ball,’ and then the person is asked to participate in a task to complete words, they are more likely to respond with the word ball to the presentation of the word bowl than if they had not previously seen that word in the original list. Thus, the priming capacity can affect the choice of a particular word on a test to complete words, even long after conscious recollection of the primed words has been forgotten.

Another context where this can be seen is in asking a participant to identify an image from a small fragment. The participant is shown a larger portion of the image over time, giving them the ability to identify the image at the end. The participant will take longer to identify the image if it is the first time he/she sees it. But if he/she already saw it in a previous trial, he/she takes less time (Kolb and Whishaw, 2003).

## Future Directions

In spite of recent progress, a number of important questions remain to be tackled.

Many of these questions have to do with molecular processes of memory consolidation, retrieval, and decay. Take, for instance, the processes underlying the LTP of synaptic strength among neurons of the hippocampus (Dong et al., 2015). In their review, Hardt et al. (2013) point out that while the “molecular processes involved in establishing LTP have been characterized well, the decay of early and late LTP is poorly understood.” One possibility that has recently been suggested is that LTP decay is mediated by AMPAR endocytosis, which in turn implies that inhibition of this process could preserve LTP and help to prevent memory loss (Dong et al., 2015).

Other recent work shows the critical role of dopamine as a signal that promotes the stable incorporation of novel information into long-term hippocampal memory (Otmakhova et al., 2013). Indeed, dopamine neurons can be activated by novelty in the absence of reward and it is thought that this activation occurs via a polysynaptic pathway that runs from the hippocampus to the dopamine cells of the VTA. However, many aspects of this process remain unclear (see Otmakhova et al., 2013).

Also requiring further investigation are the molecular processes involved in the regulation of protein synthesis related to memory. It is now thought that protein synthesis is not only involved in the consolidation of new memories, but must also be used to re-consolidate memories that have been degraded or “destabilized” as a result of retrieval. In a recent article, Jarome et al. (2016) indicate that CaMKII controls the re-consolidation process through the regulation of proteasome activity. Another mechanism for the regulation of protein synthesis involves MicroRNAs (miRNAs), a class of short, non-coding RNAs. By regulating components of pathways required for learning and memory, miRNAs modulate the influence of epigenetics on cognition in the normal and diseased brain (Saab and Mansuy, 2014).

Another set of important questions relates to the long-standing hypothesis of “Hebbian learning”—the strengthening of synapses between neurons with correlated activity—and its role in memory. At the same time that we are learning more about the mechanisms involved in Hebbian plasticity, we are also learning about how these mechanisms are complemented by synaptogenesis and neuromodulatory processes. Recent research has shown that synaptogenesis is not only important during development, but also plays a central role in associative learning and memory. Synaptogenesis can be triggered by neuron–astrocyte or neuron–neuron contact, and mediated by cell-adhesion proteins including neurexin/neuroligin, Eph receptors, and cadherins, which activate intracellular signaling pathways involving cofilin, GTPases, and other proteins (for a review see Nelson and Alkon, 2015). Others have proposed that Hebbian processes, while important, are not sufficient for memory formation, and must be supported by the activation of neuromodulatory processes, especially in the case of associative aversive learning (Johansen et al., 2014). Another study found evidence for both Hebbian and anti-Hebbian mechanisms of synaptic plasticity, indicating that the mechanisms of learning are highly adaptable (Koch et al., 2013).

Finally, other questions relate to the special role of the amygdala in the emotional enhancement of memory consolidation. It has long been known that emotional arousal contributes to the selection and consolidation of memory. It has also been shown that previously weak or inconsequential information can be strengthened retroactively through an emotional learning experience (Dunsmoor et al., 2015). Evidence suggests that it is the amygdala that is most responsible for the enhancement of memory (de Voogd et al., 2016). As suggested by a review, these developments “will likely lead to an updated view of the amygdala as a critical nexus within large-scale networks supporting different aspects of memory processing for emotionally arousing experiences” (Hermans et al., 2014). Moreover, this research is likely to have important implications for the treatment of psychological disorders (Beckers and Kindt, 2017).

## Conclusion and Glossary

**FIGURE 3:**
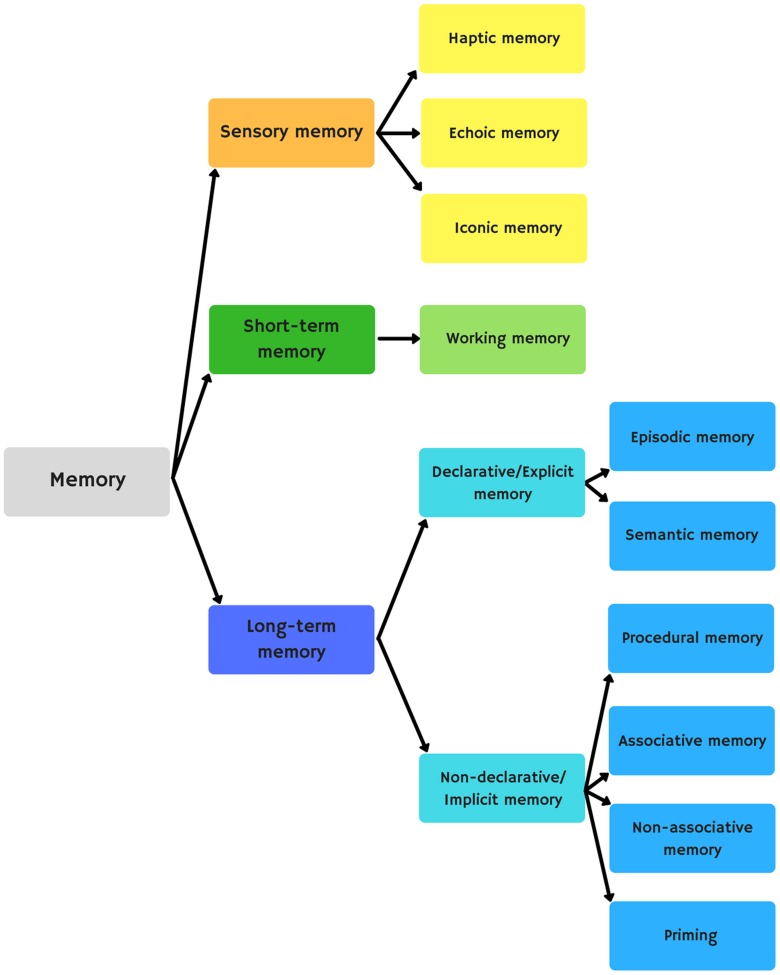
Memory classification.

There are three main forms of memory: sensory memory, short-term memory, and long-term memory (**Figure 3**). Sensory memory refers to the retention of information coming from the senses. Short-term memory refers to information processed in a short period of time. Working memory performs this processing. Working memory consists of four elements that process information: the central executive (attention control), the visuospatial sketchpad (creates and maintains a visuospatial representation), the phonological buffer (stores and consolidates new words), and the episodic buffer (stores and integrates information from different sources). Long-term memory allows us to store information for long periods of time. This information may be retrieved consciously (explicit memory) or unconsciously (implicit memory). Explicit memory consists of episodic memory (time-related events) and semantic memory (concepts and meanings). Implicit memory has, in turn, procedural memory (motor and executive skills), associative memory (classical and operant conditioning), non-associative memory (sensitization and habituation), and priming (a primary stimulus influencing a secondary one).

Finally, the following glossary includes commentary about the terminology that, in our opinion, is essential for an introductory overview, enabling interested students and professionals to effectively approach the latest memory-related discoveries. This commentary is not intended as an exhaustive definition, but rather collects relevant information to situate the reader within a complex panorama.

*Associative memory*: refers to the storage and retrieval of information resulting from an association (i.e., resulting from an association with other information). Two types of conditioning are involved in its acquisition: classical conditioning and operant conditioning. Classical conditioning is a kind of associative learning between stimuli and behavior, and operant conditioning is a form of learning in which new behaviors develop in terms of their consequences.

*Conceptual short-term memory/episodic buffer*: This is a temporary storage system capable of integrating information from different sources that is probably controlled by the central executive. It is episodic in that it has episodes in which information is integrated through space and, potentially, extended through time.

*Echoic memory*: sensory memory that receives and processes auditory information.

*Episodic memory*: “involves the ability to learn, store, and retrieve information about unique personal experiences that occur in daily life. These memories typically include information about the time and place of an event, as well as detailed information about the event itself” (Dickerson and Eichenbaum, 2010).

*Explicit/declarative memory*: refers to conscious memories of previously stored experiences, facts and concepts that are verifiable through a verbal reporting of them (Tulving, 1972).

*Haptic memory*: sensory memory that receives and processes information from the sense of touch.

*Iconic memory*: visual-sensory memory that receives and processes visual stimuli.

*Implicit/non-declarative memory*: this encompasses all unconscious memories, as well as certain abilities or skills. There are four types of implicit memory: procedural, associative, non-associative, and priming memory.

*Long-term memory*: “refers to the unlimited, continuing memory store that can hold information over lengthy periods of time, even for an entire lifetime. Long-term memory is mainly preconscious and unconscious. Information in long-term memory is to a great extent outside of our awareness, but can be called into working memory to be used when needed. Some of this information is easy to recall, but some is much more difficult to access” (Brodziak et al., 2013).

*Non-associative memory*: refers to newly learned behavior due to repeated exposure to a single stimulus. The new behavior can be classified into two processes: sensitization and habituation.

*Perceptual memory*: memory acquired through the senses. It includes a lot of individual experience; it ranges from the simplest forms of sensory memory to the most abstract knowledge.

*Priming*: an effect whereby exposure to certain stimuli influences the response to subsequently presented stimuli.

*Procedural memory*: a memory area involved in remembering executive and motor skills necessary to perform a task. It is an executive system that guides activity and usually works on an unconscious level. When necessary, procedural memories are automatically retrieved for use in the implementation of integrated procedures related to motor and intellectual skills.

*Semantic memory*: refers to the memory of meanings, interpretations and concepts related to facts, information and general knowledge about the world. Semantic memory gives meaning to words and phrases that would otherwise be meaningless and allows for learning based on past experience (Kolb and Whishaw, 2003).

*Sensory memory*: “Sensory memory is the capacity for briefly retaining the large amounts of information that people encounter daily” (Siegler and Alibali, 2005).

*Short-term memory:* is the ability to keep a small amount of information available for a short period of time. “Short-term memory should be distinguished from working memory, which refers to structures and processes used for temporarily storing and manipulating information. The relationship between short-term memory and working memory is presented variously by different theories. The notion of working memory is broader and more general because it refers to structures and processes used for temporarily stored and manipulated information” (Brodziak et al., 2013).

*Working memory*: “The term working memory refers to a brain system that provides temporary storage and manipulation of the information necessary for such complex cognitive tasks as language comprehension, learning and reasoning” (Baddeley, 1992).

*Visual memory*: constituted by iconic memory, visual short-term and long-term memory.

*Visual short-term memory/visuospatial sketchpad*: sketchpad’s main function is to create and maintain a visuospatial representation that persists through the irregular form found in eye movement and that characterizes our exploration of the visual world (Luck, 2007).

## Author Contributions

FG drafting of sensory and short-term memory; bibliographical review; introduction and conclusion. Revising critically and final approval of the version to be published. EC drafting of long-term memory (declarative and non-declarative); bibliographical review.

## Conflict of Interest Statement

The authors declare that the research was conducted in the absence of any commercial or financial relationships that could be construed as a potential conflict of interest.
